# Structure–Property Relationships of Boron Nitride-Reinforced Glass Fiber/Epoxy Laminated Composites

**DOI:** 10.3390/polym18030372

**Published:** 2026-01-30

**Authors:** Sakine Kıratlı, Selçuk Özmen

**Affiliations:** 1 Department of Mechanical Engineering, Faculty of Engineering, Cankiri Karatekin University, 18100 Cankiri, Turkey; 2 Institute of Science and Technology, Faculty of Engineering, Cankiri Karatekin University, 18100 Cankiri, Turkey

**Keywords:** glass fiber/epoxy laminates, hexagonal boron nitride (h-BN), mechanical performance, thermal stability, electrical insulation

## Abstract

Advances in modern industry largely depend on the development of high-performance materials. In this study, the influence of hexagonal boron nitride (h-BN) filler on the performance of glass fiber/epoxy laminates was systematically investigated. Composites containing h-BN with different particle sizes (65–75 nm and 790 nm) and contents (0.2 and 0.4 wt.%) were fabricated, and their mechanical (tensile, in-plane shear, hardness, impact), thermal (Differential Scanning Calorimetry, DSC), electrical (volume resistivity), and spectroscopic (Fourier Transform Infrared Spectroscopy, FTIR) properties were examined. The results demonstrated that specimens with 65–75 nm h-BN at 0.2 wt.% exhibited the highest tensile and shear strengths, whereas those with 790 nm h-BN at 0.4 wt.% showed superior impact resistance and hardness. DSC analyses revealed that h-BN addition increased the glass transition temperature (Tg), while FTIR confirmed interfacial interactions between h-BN and the epoxy matrix. Electrical measurements indicated that h-BN preserved the insulating nature of the composites, with only limited reductions in resistivity observed at higher contents of larger particles due to morphological effects. Overall, these findings highlight that h-BN filler enhances load transfer efficiency, thermal stability, and mechanical reliability, offering significant potential for applications requiring multifunctional performance, such as aerospace, marine, and electrical and electronic insulation systems.

## 1. Introduction

Glass fiber-reinforced thermoset polymer composites (GFRPs) are widely used in aerospace, automotive, marine, wind turbine, and sporting goods applications [1]. These materials overcome several disadvantages of conventional counterparts by offering low weight, high stiffness, and superior strength. Nevertheless, previous studies have highlighted the need to further improve their responses to diverse mechanical, dynamic, thermal, and electrical contents. To this end, various filler strategies have been explored to modify polymer matrices, aiming to enhance stress transfer, delay crack initiation and propagation, and strengthen interfacial adhesion [2,3].

Numerous nanoparticles including carbon nanotubes [4], nanoclays [5], graphene nanoplatelets [6], nanofibers [7], and boron nitride (BN) [8] have been incorporated into polymer composites with varying degrees of success. Among these, BN stands out due to its availability and unique property profile. BN is a super-hard ceramic material composed of boron and nitrogen atoms [9]. It exists mainly in two crystalline forms: cubic BN (c-BN) and hexagonal BN (h-BN). Hexagonal BN exhibits outstanding features such as refractory stability at high temperatures, superior thermal shock resistance, high thermal conductivity, electrical insulation, chemical inertness, lubricity, and ease of machining [10]. These properties make h-BN attractive for applications in metallurgy, high-temperature molding lubricants, and as an insulating material in the electronics industry [10]. In addition, its durability and performance are well recognized in industrial sectors such as cutting tools, abrasive products, and other high-temperature applications [11]. Owing to its versatile nature, BN continues to inspire new and promising applications across both mechanical and electronic systems.

A wide range of studies have explored the effects of BN on the thermal and electrical performance of glass fiber/epoxy composites. For instance, Shi et al. [12] demonstrated that incorporating heterostructured spherical BN and BN nanosheets markedly enhanced both thermal conductivity and electrical insulation. At a 15 wt.% BN content, the in-plane and through-plane thermal conductivities reached 2.75 and 1.32 W/m·K, respectively—approximately 12.5- and 6-fold improvements compared to neat epoxy. The hybrid fillers also increased Tg to 167 °C, improving thermal reliability. Tang et al. [13] reported that functionalized glass fiber/spherical BN/epoxy composites exhibited significant enhancements at low filler contents, with 1 wt.% BN increasing dielectric strength to 27 kV/mm and 20 wt.% BN achieving an in-plane thermal conductivity of 3.55 W/m·K (~15-fold increase), while maintaining Tg. Li et al. [14] prepared APTES-modified BN/polyvinyl alcohol-supported heterostructured composites that demonstrated notable improvements in both thermal and dielectric performance, achieving a thermal conductivity of 0.669 W/m·K (~3× higher than neat epoxy) and retaining 37.8% char yield at 800 °C. Nanda and Satapathy [15] showed that BN incorporation enhanced thermal conductivity by 46% in epoxy/h-BN/human hair fiber hybrids, while the hair fibers improved Tg and dimensional stability. However, 20 wt.% hair fiber significantly increased dielectric loss, limiting high-frequency applications. Zhang et al. [16] used silica-sol-functionalized glass fibers with spherical BN, reporting in-plane and through-plane conductivities of 2.37 and 1.07 W/m·K (11- and 5-fold increases) and improved dielectric strength (22.3 kV/mm) with preserved thermal stability up to 197.3 °C.

Beyond glass fiber systems, BN has also been shown to influence natural fiber composites. Eryılmaz et al. [17] reported that h-BN addition to flax/epoxy composites led to reductions in tensile and shear properties at 1.5 wt.% due to particle agglomeration and weak interfacial bonding. In contrast, Agrawal et al. [18] demonstrated that hybrid epoxy/h-BN/sisal fiber composites exhibited a 36.5% increase in tensile strength at 10 wt.% BN + 3 wt.% sisal, while 40 wt.% BN improved microhardness nearly 8.5-fold. Venkatesh [19] further observed that LDPE/ramie fiber composites containing 9 wt.% BN showed a 51% increase in tensile strength and 20% increase in hardness.

BN fillers have also been studied in carbon fiber composites. Muralidhara et al. [20] found that adding micro-BN to carbon fiber/epoxy composites improved tensile strength (up to 725 MPa at 1 wt.% BN), Shore-D hardness, and thermal stability, although impact resistance decreased at higher BN contents. Rahmat et al. [21] reported that 1 wt.% BN nanotubes enhanced interlaminar fracture properties, increasing impact energy by 18% and raising Tg. Uzay [22] demonstrated that adding nano-BN to carbon fiber composites improved tensile strength (20% increase), impact toughness (42% increase), decomposition temperature (351 °C), and Tg (~144 °C). Similarly, 2–3 wt.% c-BN yielded the greatest improvements in both tensile and impact properties, with a 14 °C increase in Tg.

More recently, functionalized BN has been used to achieve stronger interfacial interactions. Gou et al. [23] employed diazonium-functionalized BN nanosheets deposited via layer-by-layer assembly on glass fibers, resulting in a 23.5% increase in tensile strength, AC dielectric strength of 42.1 kV/mm, and thermal conductivity of 2.12 W/m·K (~7× improvement over neat composites). Özbaş et al. [24] reported that silanized nano-BN-coated carbon fiber/PA12 composites exhibited a 17.5% increase in tensile strength, higher hardness, and a significant reduction in electrical resistance (from 5.5 kΩ to 1.9 kΩ). DSC analyses confirmed that APTES-modified h-BN increased Tg.

Collectively, these studies demonstrate that BN, in different morphologies and at various contents, can significantly enhance the mechanical, thermal, and electrical performance of composites. However, systematic investigations directly comparing BN types and concentrations in glass fiber/epoxy laminates remain limited. The present study addresses this gap by evaluating the mechanical, thermal, and electrical properties of E-glass/epoxy composites reinforced with two particle sizes (65–75 nm and 790 nm) and two contents (0.2 and 0.4 wt.%). The damage behavior and mechanisms were characterized through SEM and FTIR analyses, providing insights into interfacial interactions and failure evolution. The novelty of this work lies in the comparative, multi-property evaluation of different BN morphologies within the same composite system, thereby filling a critical gap in the literature and highlighting BN’s potential to optimize multifunctional performance in structural composites.

## 2. Materials and Test Methods

### 2.1. Materials

The composite system investigated in this study consisted of an epoxy-based matrix, E-glass fibers, and hexagonal boron nitride (h-BN) nanoparticles with two different particle sizes. The epoxy resin (F-1564, viscosity 1250–1450 mPa·s at 25 °C, density 1.1–1.2 g/cm^3^ at 25 °C) and curing agent (F-3487, viscosity 30–70 mPa·s at 25 °C, density 0.98–1.0 g/cm^3^ at 25 °C) were supplied by Fibermak (Izmir, Türkiye). According to the supplier’s specifications, the recommended mixing ratio of resin to hardener was 100:34 by weight. The E-glass fibers, also obtained from Fibermak, were unidirectional continuous fibers with an areal weight of 330 g/m^2^. The specifications and typical mechanical properties of the E-glass fiber fabric used as reinforcement in this study are summarized in Table 1.

The h-BN nanoparticles used as the reinforcing phase were procured from Nanografi (Ankara, Türkiye) in two particle sizes: 65–75 nm and 790 nm. These nanoparticles were incorporated into the epoxy matrix at weight fractions of 0.2 and 0.4 wt.%. To achieve a uniform dispersion of nanoparticles within the resin, a combination of mechanical stirring and ultrasonication was employed. Mechanical mixing was performed at 800 rpm for 30 min using a Velp Ohs 20 device (Valera, MB, Italy). Following this, mixing was performed for 30 min using the Sonics Vibra Cell device (Newtown, CT, USA) with a 9 s on, 2 s off setting. To prevent excessive heating during mixing, the outside of the beaker was supported by an ice bath and temperature control was ensured using a thermocouple. Finally, a hardener was added to the nanoparticle mixture and stirred for 10 min using a mechanical stirrer.

The nanoparticle-reinforced laminated composites were fabricated using the hand lay-up method followed by vacuum bagging. For composite fabrication, a nylon film was first laid on a rigid flat surface, and its edges were sealed with leak-proof tape to ensure vacuum integrity. A vacuum line was established on one side using a spiral hose–valve assembly. E-glass fiber fabrics with specified dimensions and orientations were then sequentially placed in the open area and impregnated layer by layer with the epoxy/boron nitride/hardener mixture using a brush. After completing the lay-up, a release fabric was placed over the laminate, followed by a perforated nylon film and a vacuum blanket to allow uniform vacuum distribution and resin bleed. The entire assembly was subsequently covered with a vacuum bagging film and sealed. Vacuum was then applied to consolidate the laminate and remove entrapped air. The laminated composites were cured under vacuum at room temperature for 48 h. After curing, the vacuum bagging materials were removed, and the consolidated laminates were cut into test specimens according to the relevant testing standards.

Laminates for tensile testing were prepared with four plies, those for shear testing with sixteen plies, and those for compressive, impact, hardness, electrical, and thermal analyses with ten plies. The codes assigned to the fabricated composite specimens are summarized in Table 2.

### 2.2. Characterizations

**Figure 1:**
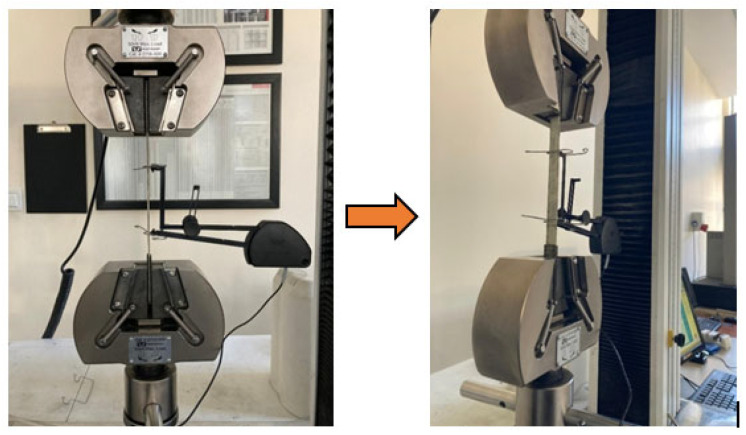
Tensile test setup and specimen configuration for E-glass/epoxy laminated composites tested in accordance with ASTM D3039.

Testing tests play a critical role in evaluating the mechanical properties of composite materials. In this study, tensile tests were conducted on composite specimens with dimensions of 250 × 15 × 2 mm in accordance with ASTM D3039 [25]. The tests were performed along the fiber direction ([0]_4_ oriented laminates) using an Instron universal testing machine (Norwood, MA, USA) with a load capacity of 50 kN. To minimize local stress concentrations at the gripping regions, each specimen was fitted with end-tabs. The end-tabs were 80 mm in length, and the grip pressure was controlled to remain below 0.5 MPa during testing. A constant crosshead speed of 2 mm/min was applied, and a preload of 10 N was imposed before clamping the specimen into the grips. Throughout the test, stress–strain data were continuously recorded by the testing machine at a data acquisition rate of 50 Hz until specimen failure (Figure 1).

**Figure 2:**
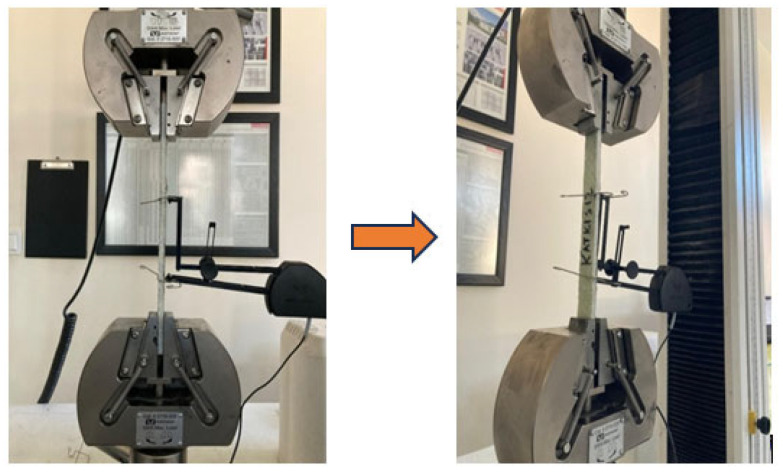
In-plane shear test setup and specimen configuration of E-glass/epoxy laminated composites tested in accordance with ASTM D3518.

The shear behavior of the composites was evaluated in accordance with ASTM D3518 [26], which provides a standardized method for testing specimens with a ±45° fiber orientation. The test specimens were prepared with dimensions of 250 × 25 × 7 mm and fiber oriented at ±45° ([0/90]_4S_ oriented laminates). Shear tests were performed using an Instron universal testing machine with a 50 kN load capacity at a constant crosshead speed of 1 mm/min. Stress–strain curves were continuously recorded in real time using an extensometer and a load cell. A preload of 5 N was applied, and each test was continued until a strain level of 5% was reached (Figure 2).

**Figure 3:**
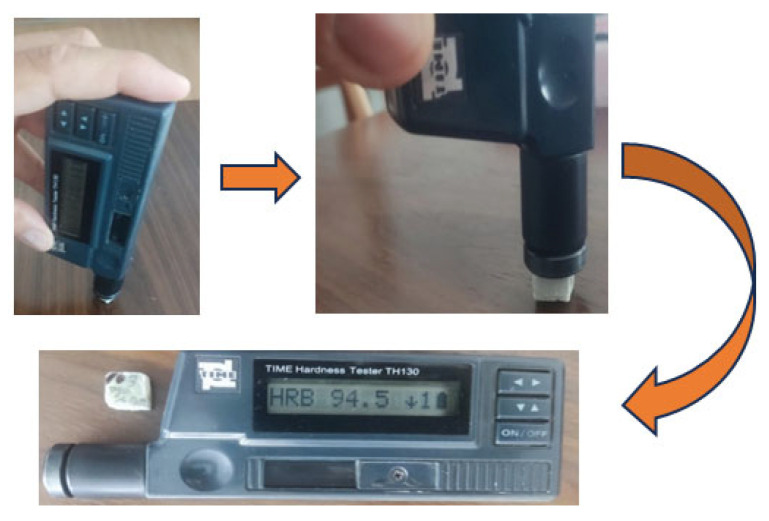
Rockwell hardness testing setup for E-glass/epoxy laminated composite specimens conducted in accordance with ASTM D785.

The hardness of the composite specimens was measured in accordance with ASTM D785 [27] using the Rockwell B (HRB) scale. Tests were carried out on [0]_10_ oriented laminates using a calibrated Rockwell hardness tester. For each specimen, measurements were taken at a minimum of five different locations, and the average value was reported as the hardness result (Figure 3). Care was taken to ensure that specimen surfaces were smooth and well-polished, with appropriate preparation performed to eliminate the influence of surface irregularities or layer inconsistencies on the test results. The obtained hardness values were comparatively analyzed as a function of filler content and particle size to evaluate the effect of nanostructured filler on the composites’ resistance to deformation.

**Figure 4:**
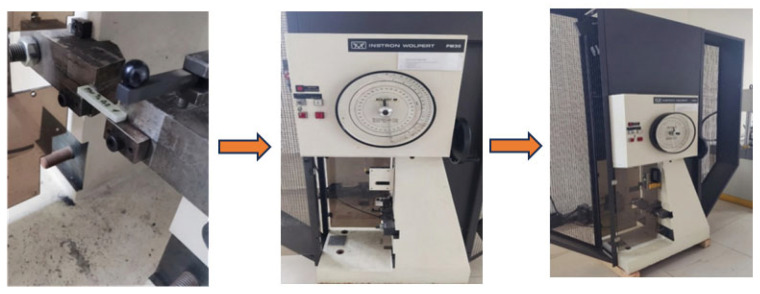
Izod impact test setup and specimen configuration for E-glass/epoxy laminated composites tested in accordance with ASTM D256.

Impact tests were conducted in accordance with ASTM D256 [28] on unnotched [0]_10_ oriented specimens with dimensions of 100 × 25 × 7 mm. The use of unnotched specimens enabled a clear observation of the viscoelastic response. The specimen surfaces were machined to a surface roughness of Ra ≤ 1.6 μm. The impact point was marked at a distance of 62 mm. The tests were performed using an Instron Wolpert PW 30 pendulum impact tester with a capacity of 15 J, and the pendulum was set at an angle of 150° (Figure 4).

The impact strength (IS) was calculated using Equation (1):

IS = *E*/(*b* × *d*) (1)

where

- *E* is the absorbed energy (J);
- *b* is the specimen width (mm);
- *d* is the specimen thickness (mm).

**Figure 5:**
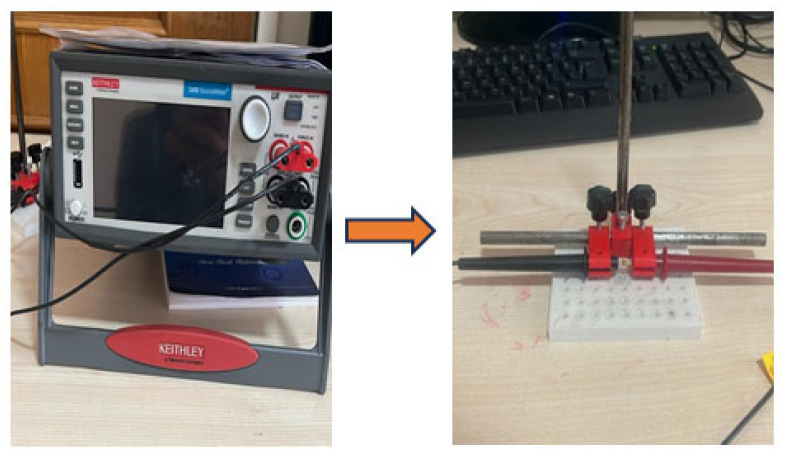
Electrical conductivity measurement setup for E-glass/epoxy laminated composite specimens conducted in accordance with ASTM D257.

Electrical properties of the composite specimens were assessed through volume and surface resistivity measurements, which play a critical role in evaluating insulating materials. ASTM D257 [29] was adopted as the standard method, as it provides a reliable approach for electrical characterization of dielectric composites. In this study, the electrical conductivity of h-BN reinforced laminated composites ([0]_10_ oriented specimens) was systematically evaluated using a high-precision source measurement unit (Keithley 2450 SourceMeter^®^, Beaverton, OR, USA). The measurements were performed with the four-probe (Kelvin) method under a DC voltage of 100 V (Figure 5). The specimens were molded into dimensions of 10 × 10 × 7 mm, and their surfaces were polished to a surface roughness of Ra ≤ 0.8 µm. To improve electrode contact and minimize contact resistance, the electrode interfaces were coated with a silver-based conductive paint.

Differential scanning calorimetry (DSC) analyses were carried out on both neat and BN-reinforced specimens in accordance with ASTM D3418 [30]. Standard operational procedures of the DSC instrument were followed throughout the experiments. A specimen mass of 3.7 mg was used, and measurements were conducted against an empty reference pan. According to the DSC protocol, each specimen was heated from an initial temperature of 30 °C up to 500 °C in ramp mode at a heating rate of 20 °C/min. During this process, temperature was continuously increased while variations in heat flow were precisely recorded. The instrument was programmed to collect data at intervals of 0.5 s. At the end of the heating cycle, the specimens were held isothermally at 500 °C for 5 min to evaluate thermal stability and possible degradation phenomena. To ensure data reliability, the instrument’s recording function was activated, and the entire heat flow profile was digitally stored for subsequent analysis.

### 2.3. Morphological Analysis

The fracture surface morphologies of neat and BN-reinforced specimens after shear testing were examined using scanning electron microscopy (SEM). Before SEM observations, the specimen surfaces were sputter-coated to ensure surface conductivity. In this study, palladium sputter coating was employed instead of conventional gold coating, as palladium provides superior uniformity and forms a thinner conductive layer. This approach enabled high-resolution imaging under high vacuum conditions and preserved fine surface details, particularly for specimens with low inherent conductivity. All microstructural imaging and analyses were conducted using a high-resolution Sigma 300 VP SEM instrument (Oberkochen, Germany).

Fourier transform infrared spectroscopy (FTIR) was performed to investigate the chemical structure and functional groups of the composite specimens. Measurements were carried out using a Bruker Tensor II spectrometer (Billerica, MA, USA) equipped with an ATR accessory containing a diamond crystal. This technique allows direct contact with the specimen surface to obtain spectra and requires no additional specimen preparation. The resulting spectra were analyzed to identify the characteristic absorption bands of the epoxy matrix, to detect possible interaction regions associated with BN filler, and to evaluate the chemical compatibility of the filler with the polymer chains.

## 3. Results and Discussion

### 3.1. Tensile Testing

In this study, composite laminates with a [0]_4_ lay-up configuration were used. Tensile tests were performed along the fiber direction for both neat and BN-reinforced specimens. From the tests, the tensile strength, elongation at break, and Young’s modulus were determined. Each test was repeated five times, and the mean values obtained from the experiments are summarized in Table 3.

Table 3 shows the average tensile strength values of neat and BN-reinforced E-glass/epoxy composites. The highest tensile strength was measured as 297 MPa for the specimen containing 0.2 wt.% of 65–75 nm BN, indicating that an appropriate particle size and content effectively enhance the fiber–matrix interfacial interaction. In contrast, the addition of the same particle size at 0.4 wt.% resulted in a pronounced decrease in tensile strength (122.4 MPa), suggesting that higher contents promote agglomeration and thereby weaken the structural integrity. The lowest tensile strength was recorded as 104.9 MPa for the composite reinforced with 0.2 wt.% of 790 nm BN, which suggests a detrimental effect of increasing BN particle size on the mechanical performance.

Table 3 presents the average Young’s modulus values of neat and BN-reinforced specimens. The highest modulus, 24.8 GPa, was obtained for the composite containing 0.4 wt.% of 790 nm BN, indicating that in certain cases, larger particles can contribute to higher stiffness. This was followed by the specimen reinforced with 0.2 wt.% of 65–75 nm BN, which exhibited a modulus of 20.5 GPa. In contrast, the neat specimen showed a modulus of only 4.6 GPa. These comparisons demonstrate that BN filler substantially improved the elastic properties of the polymer matrix. Nevertheless, the balance between filler size and content plays a critical role in elastic performance, as some higher contents (e.g., 0.4 wt.% of 65–75 nm BN) resulted in decreases in modulus.

**Figure 6:**
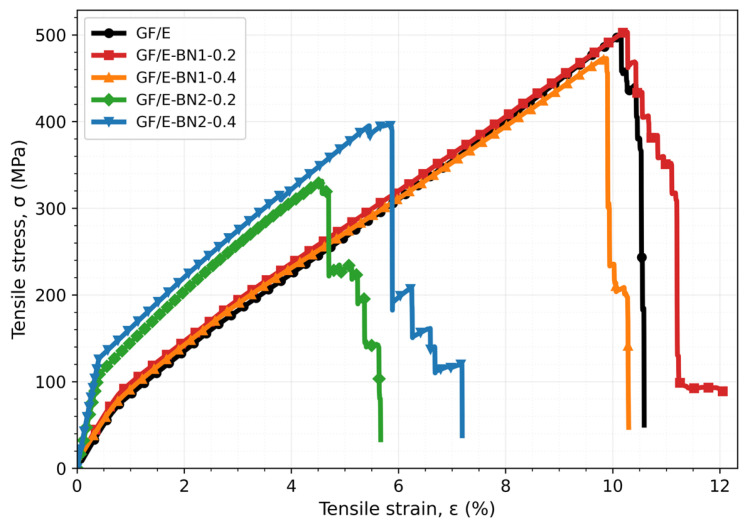
Tensile stress–strain curves of neat and BN-filled E-glass/epoxy composites.

The comparison of tensile stress–strain curves (Figure 6) reveals that boron nitride (BN) particle size and reinforcement ratio directly affect the load-carrying mechanisms in E-glass/epoxy composites. The high fracture elongation observed in the neat GF/E laminate is associated with the ductile behavior of the epoxy matrix, which allows for free deformation and effective stress distribution in the inter-fiber region. The addition of BN particles with a size of 65–75 nm at a weight ratio of 0.2 wt.% contributed to a more homogeneous distribution of the applied load by providing an increase in micro-scale stiffness at the matrix–fiber interface, resulting in an increase in tensile stress levels. Increasing the BN content to 0.4 wt.% with the same particle size limited the deformation capacity due to the restriction of nanoparticle movement between epoxy chains and the formation of local hard regions, leading to a decrease in the final unit strain. In composites containing larger BN particles (790 nm), the reduction in particle-matrix interface area and the formation of stress concentrations around the particles led to the emergence of early damage initiation zones, especially at high loading ratios. These findings demonstrate that composite performance under tensile loading is strongly dependent not only on the amount of reinforcement but also on the distribution of nanoparticles within the matrix and the effectiveness of interface interactions.

**Figure 7:**
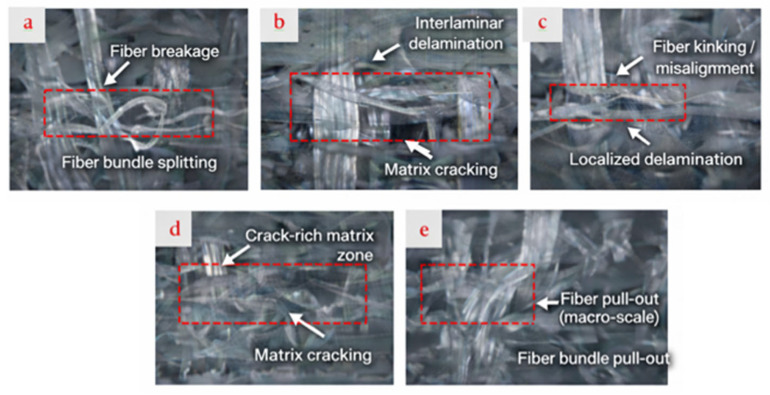
Optical microscope images of damaged tensile specimens: (**a**) GF/E, (**b**) GF/E-BN1-0.2, (**c**) GF/E-BN1-0.4, (**d**) GF/E-BN2-0.2, and (**e**) GF/E-BN2-0.4.

Optical microscopy images of the tensile-damaged specimens are shown in Figure 7. The neat GF/E composite exhibited a predominantly brittle fracture behavior, which can be attributed to extensive matrix cracking and relatively weak fiber–matrix interfacial bonding. In the GF/E-BN1-0.2 specimen, the incorporation of 65–75 nm BN particles led to improved interfacial integrity, as indicated by enhanced fiber encapsulation within the matrix and increased matrix deformation. In contrast, increasing the BN content to 0.4 wt.% (GF/E-BN1-0.4) resulted in particle agglomeration, which generated localized stress concentrations at the interface and adversely affected the tensile response. For the GF/E-BN2-0.2 specimen, insufficient interfacial bonding between the fibers and the matrix promoted crack initiation and propagation. Conversely, the GF/E-BN2-0.4 composite demonstrated improved matrix continuity around the fibers, accompanied by evident fiber pull-out and pronounced matrix deformation. These features indicate a transition toward a more ductile failure mechanism under tensile loading.

The tensile response of the laminates is governed by a clear particle-size/content interplay. Across all conditions, incorporating 65–75 nm h-BN at 0.2 wt.% yields the highest strength, while increasing the content to 0.4 wt.% or switching to 790 nm particles diminishes performance. These trends indicate that fine particles at low loading promote efficient stress transfer at the fiber–matrix interface, whereas higher loadings and/or larger particles reduce the effective load-bearing area through particle clustering.

Mechanistically, the improvement at low, nanoscale loading is consistent with (i) more homogeneous dispersion within the epoxy, (ii) increased specific interface for shear-lag transfer to the glass fibers, and (iii) suppression of early matrix cracking. The deterioration at higher loading/large size aligns with agglomeration-induced stress concentrations that localize damage and trigger premature crack initiation. Optical microscopy of the fractured specimens corroborates these interpretations: the best-performing condition shows deeper fiber embedding and pronounced matrix deformation, whereas agglomerated conditions display interfacial debonding and easier crack advance. These microstructural signatures are congruent with the measured strength/modulus trends.

The observations here agree with prior reports that modest BN additions enhance interfacial adhesion and tensile behavior, while excessive contents or unfavorable morphologies curtail the benefit due to clustering and weak links [12,13]. Taken together with the thermal/FTIR evidence presented later (Tg upshifts and epoxy–BN interaction bands), the tensile improvements at low nanoscale loading are plausibly rooted in both physical confinement of chain mobility and interfacial interactions that sharpen stress transfer.

### 3.2. Shear Testing

In this study, ±45° specimens were prepared from composite laminates with a [0/90]_4S_ lay-up configuration. Shear properties were determined through tensile testing for both neat and BN-reinforced specimens. From the tests, the in-plane shear strength, shear strain at failure, and shear modulus were obtained. Each test was repeated five times, and the mean values derived from the experiments are summarized in Table 4.

Table 4 compares the average shear strength values of neat and BN-reinforced E-glass/epoxy composites. The highest shear strength, 89.1 MPa, was obtained for the specimen containing 0.2 wt.% of 65–75 nm BN. This result indicates that an appropriate filler size and content can enhance fiber–matrix interfacial interactions and significantly improve shear resistance. However, increasing the filler content to 0.4 wt.% with the same particle size reduced the shear strength to 73.0 MPa. This decrease may be attributed to particle agglomeration at higher contents, which can induce stress concentrations within the structure. For composites prepared with 790 nm BN, the shear strength values were 64.0 MPa at 0.2 wt.% and 63.1 MPa at 0.4 wt.%, indicating the limited contribution of larger particles to shear performance.

The average shear modulus values of the composite specimens are presented in Table 4. The highest shear modulus, 7.4 GPa, was obtained for the composite reinforced with 0.2 wt.% of 65–75 nm BN. This result indicates that the elastic response of the matrix can be significantly enhanced by incorporating a small amount of fine BN particles. In contrast, the neat composite exhibited a modulus of only 5.2 GPa, clearly highlighting the positive influence of BN filler on elastic properties. For composites containing 790 nm BN, the modulus values were close to each other (6.5 and 6.6 GPa), suggesting that larger particles provide only a limited contribution to stiffness enhancement. The 65–75 nm BN composite with 0.4 wt.% content showed a moderate performance with a modulus of 6.0 GPa. Overall, the results indicate that the optimum shear modulus performance was achieved with a low content of fine BN particles.

**Figure 8:**
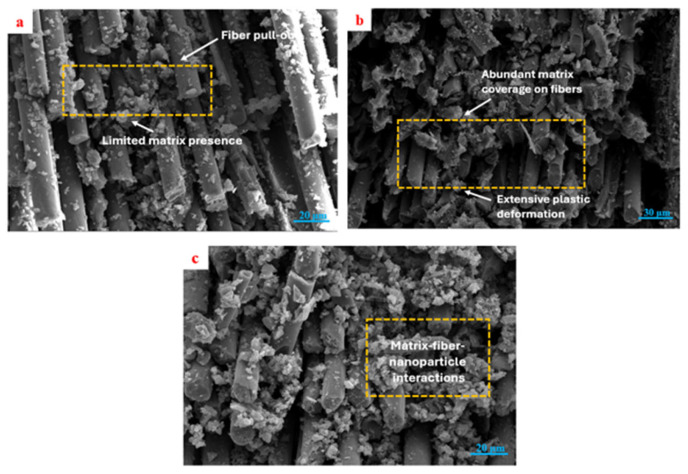
SEM images of damaged shear specimens: (**a**) GF/E, (**b**) GF/E-BN1-0.2, and (**c**) GF/E-BN2-0.4.

SEM observations of damaged shear specimens are shown in Figure 8. When evaluated together with the mechanical data, the neat GF/E specimen exhibited weak matrix adhesion on the fiber surfaces and easy fiber detachment, which correlated with its low shear strength and limited strain values. In the GF/E-BN1-0.2 specimen, abundant matrix residues around the fibers and pronounced deformation traces revealed strong fiber–matrix interfacial interactions, consistent with the high shear strength and strain values obtained. For the GF/E-BN2-0.4 specimen, matrix adhesion on the fiber surfaces and fiber pull-out mechanisms were evident, indicating improved interfacial bonding compared to the neat composite. Although its shear strength was lower than that of BN1-0.2, the shear modulus and strain values suggested a more balanced performance. Overall, BN filler was found to markedly enhance the shear properties when used at small particle sizes and low contents, whereas higher contents or larger particles, despite the risk of agglomeration, still provided better interfacial bonding than the neat composite.

Shear data reveal the same morphology–content balance but with added sensitivity to interface quality. The 65–75 nm, 0.2 wt.% condition provides the highest in-plane shear strength and the top-tier shear modulus, evidencing strong fiber–matrix coupling and efficient load transfer under ±45° deformation. Elevating the nanoscale content to 0.4 wt.% reduces strength (consistent with agglomeration-driven stress concentrators), while 790 nm particles yield intermediate strengths and moduli—suggesting that larger particles stiffen the matrix to a degree but are less effective at strengthening the interface.

Fractography after shear confirms these interpretations: neat laminates show facile fiber pull-out and limited matrix adherence; the best-performing nanoscale/low-content condition exhibits abundant matrix remnants on fibers and extensive plastic deformation zones; the higher-content or larger-particle cases display mixed features, including evidence of pull-out and localized debonding. Such signatures map directly onto the measured strength–modulus–strain combinations and point to interfacial integrity as the primary lever for shear performance.

These outcomes are consonant with the literature showing that low BN nanotube/nanosheet contents can markedly elevate interlaminar or in-plane shear properties via interface reinforcement, whereas higher contents compromise performance through particle coalescence [21,23]. In concert with the DSC/FTIR findings discussed elsewhere (Tg increases and interaction bands), the shear results reinforce that fine, well-dispersed BN at low loading optimizes interfacial bonding, whereas larger particles or higher loadings should be considered mainly where impact/indentation resistance is prioritized.

### 3.3. Impact Testing

In this study, composite laminates with a [0]_10_ lay-up configuration were used. Izod impact tests were performed on both neat and BN-reinforced specimens. The tests were carried out with impact energy of 300 J, and five measurements were performed for each specimen. The impact strength values obtained from the tests are summarized as mean values in Table 5.

The strength of impact demonstrated that both the type and content of BN filler significantly influenced the toughness of the composites. As shown in Table 5, the specimen containing 0.4 wt.% of 790 nm BN exhibited the highest impact strength. This improvement can be attributed to the ability of larger BN particles to absorb impact energy more effectively. In contrast, the specimen reinforced with 0.2 wt.% of 65–75 nm BN exhibited a relatively low strength value. The neat specimen showed the lowest performance with an average impact strength of 29 MPa. In general, BN filler led to an increase in impact resistance, with the 790 nm–0.4 wt.% specimen reaching the highest value of 46.7 MPa. This enhancement is associated with the efficiency of BN in dissipating impact energy and its ability to act as a barrier against crack propagation. Although 65–75 nm BN also improved the impact strength compared to the neat composite, the effect was particularly limited at 0.2 wt.%. Increasing the filler content resulted in more pronounced improvements, especially for the 790 nm BN specimens, while the effect was less evident for the 65–75 nm BN composites. These findings emphasize the critical role of particle size and dispersion in governing the filler effect. Furthermore, the relatively high standard deviation observed in some measurements may indicate a lack of intra-specimen homogeneity or the presence of localized weak regions introduced during fabrication.

Impact performance shows a distinct dependence on particle size. The 790 nm, 0.4 wt.% configuration delivered the highest toughness, while nanoscale BN at low loading produced only marginal improvements. This trend suggests that larger particles are more effective at dissipating impact energy through mechanisms such as crack deflection, microcrack branching, and localized stress redistribution. Conversely, smaller particles primarily reinforce the fiber–matrix interface but do not provide significant barriers against crack propagation under high-strain-rate loading.

Fracture morphology supports these mechanisms: coarse BN inclusions act as obstacles that divert or blunt advancing cracks, thereby consuming more fracture energy. The limited effect of nanoscale BN on impact strength implies that interface toughening alone is insufficient to resist dynamic loads. These findings echo reports in hybrid epoxy/BN/fiber systems where micron-scale BN or high loadings improved impact resistance at the cost of stiffness [20,22]. The present results thus clarify that BN particle size is a critical design variable: nanoscale particles optimize static properties, while coarser morphologies contribute more to dynamic energy absorption. For structural components subjected to impact or vibration, larger BN fractions may therefore be strategically advantageous.

### 3.4. Hardness Testing

In this study, composite laminates with a [0]_10_ lay-up configuration were used. Hardness tests were performed on both neat and BN-reinforced specimens using the Rockwell B (HRB) scale. Each test was repeated ten times, and the average values obtained for each BN type and content are presented in Table 6.

Table 6 presents a comparative analysis of the average Rockwell B (HRB) hardness values for neat and BN-reinforced composite specimens. In particular, the addition of 790 nm BN at 0.4 wt.% enhanced the surface resistance of the material, resulting in superior hardness performance. The improvement in surface resistance can be attributed to the presence of rigid boron nitride particles within the epoxy matrix, which restricts localized plastic deformation under indentation loading. In particular, larger BN particles act as effective load-bearing inclusions, reducing matrix flow and limiting the formation of plastic zones beneath the indenter. This results in a more compact microstructure and enhanced resistance to surface penetration. Additionally, the increased constraint imposed by BN particles improves stress distribution at the surface, thereby increasing hardness values. The homogeneous dispersion of BN particles increased the resistance to deformation underload and reinforced the microstructural stability, thereby contributing to the elevated HRB values. Moreover, as the BN particle size decreased, the surface hardness was associated with the formation of a more compact and uniform structure. The relatively low standard deviations observed in some specimens indicated greater consistency across repeated measurements and suggested that the fabrication process was well controlled.

Surface hardness followed a similar particle-size-driven pattern. The 790 nm, 0.4 wt.% specimens achieved the highest Rockwell B values, indicating enhanced resistance to localized plastic deformation. This outcome can be attributed to the role of larger BN particles as rigid inclusions that constrain matrix flow and compact the microstructure. In contrast, nanoscale additions improved hardness only modestly, reflecting their stronger impact on interfacial adhesion rather than on indentation resistance.

Mechanistically, hardness enhancement arises from load transfer to stiff filler particles and the consequent reduction in plastic zones beneath the indenter. The pronounced effect of larger BN suggests that particle geometry and packing density outweigh interfacial effects under localized deformation. Similar results have been reported by Agrawal et al. [18] and Venkatesh [19], who observed marked hardness gains at higher BN contents. The present findings confirm that BN is an effective hardness-enhancing filler, with coarse particles at moderate loadings being the most beneficial.

From an application perspective, elevated hardness is relevant for wear-prone components, surface insulation panels, and tooling applications, where resistance to indentation and abrasion is crucial. Thus, while nanoscale BN remains optimal for tensile/shear reinforcement, coarse BN is more appropriate for hardness-critical uses.

### 3.5. Differential Scanning Calorimetry (DSC) Testing

In this study, 10 mg of powdered composite specimens were analyzed. Differential scanning calorimetry (DSC) tests were carried out for neat and BN-reinforced specimens in the temperature range of 30–500 °C. From these tests, values such as the glass transition temperature (Tg), melting temperature (Tm), crystallization temperature (Tc), and reaction enthalpy (ΔH) were obtained.

It is well established that the glass transition temperature (Tg) of neat epoxy systems is typically reported below 200 °C, depending on resin chemistry and curing conditions [31,32,33]. In fiber-reinforced epoxy laminates, however, the presence of rigid E-glass fibers, restricted polymer chain mobility, and interfacial interactions can significantly broaden the thermal transition region and give rise to additional endothermic or exothermic events at higher temperatures, which should not be directly interpreted as Tg [31,33]. Accordingly, the thermal events observed in the range of 250–360 °C in the present DSC results are attributed to composite-level thermal phenomena rather than the glass transition of the epoxy matrix itself.

**Figure 9:**
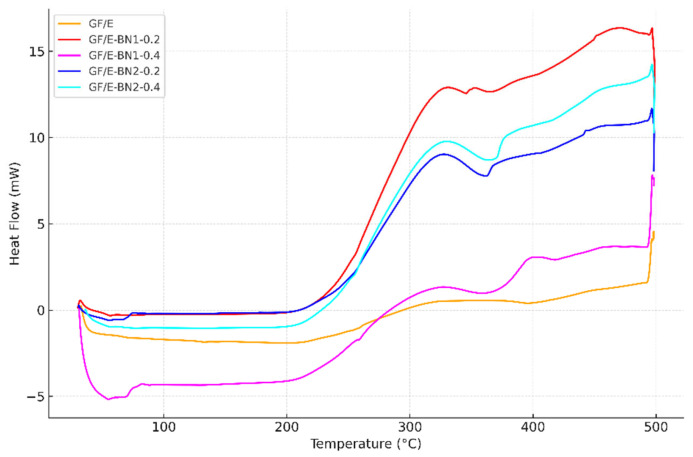
Comparison of DSC curves of neat and BN-reinforced E-glass/epoxy composites.

**Figure 10:**
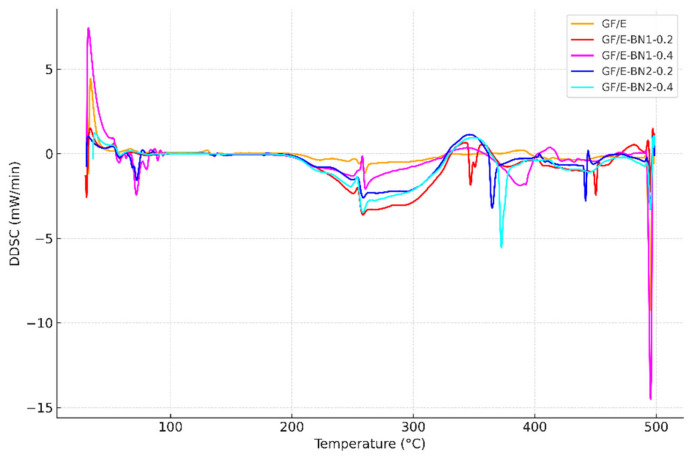
Comparison of DDSC curves of neat and BN-reinforced E-glass/epoxy composites.

The DSC (Differential Scanning Calorimetry) and DDSC (Derivative Differential Scanning Calorimetry) curves are presented in Figure 9 and Figure 10, respectively. The DDSC curve, which represents the derivative of the DSC signal with respect to temperature, allows for a clearer identification of transition regions. The sharp rise observed in the Tg region indicates that the thermal transition occurred rapidly and in a stable manner.

As shown in Figure 9, the DSC curve of the neat specimen exhibited a broad endothermic trend between approximately 250 °C and 360 °C, corresponding to the glass transition region where polymer chain mobility begins. For the BN-reinforced specimens, the Tg became more distinct and shifted along the temperature axis. In particular, the composite containing 0.4 wt.% of 790 nm BN exhibited a higher Tg, indicating that larger BN particles induced stronger physical interactions within the matrix and enhanced the thermal stability of the composite structure. Furthermore, it was observed that increasing the filler content further elevated Tg, supporting the direct influence of BN content on the thermal performance of the composites.

As shown in Figure 10, the DDSC evaluations revealed that the thermal changes during the glass transition became sharper and more pronounced. While the neat specimen exhibited a broader and less uniform distribution in the transition region, the BN-reinforced specimens showed a narrower Tg range accompanied by an increase in transition temperature. In particular, the 65–70 nm BN particles, due to their smaller size, achieved a more homogeneous dispersion within the matrix, which contributed to more stable transition behavior. Moreover, the specimen reinforced with 0.4 wt.% of 790 nm BN exhibited the sharpest and highest-temperature transition, indicating that this configuration most effectively stabilized the matrix structure.

DSC and DDSC results clearly demonstrate that h-BN incorporation enhances the thermal stability of the laminates. Neat specimens displayed a broad glass transition spanning 250–360 °C, whereas reinforced systems showed sharper transitions shifted to higher temperatures. The 790 nm, 0.4 wt.% configuration yielded the most pronounced Tg increase, indicating that coarse particles more effectively restrict polymer chain mobility and confined free volume. At the nanoscale, 65–75 nm BN also improved Tg, though the effect was less dramatic, reflecting their role in homogeneous dispersion rather than in bulk restriction.

Mechanistically, BN fillers act as physical barriers and heat-dissipating nodes, limiting chain movement and delaying the onset of thermal transitions. DDSC curves further confirmed more distinct transitions in BN-filled composites, signifying improved network stability. These findings are in line with prior reports that higher BN contents or well-structured BN networks raise Tg and suppress coefficient of thermal expansion [13,14,15].

For applications, the observed Tg improvements are critical for high-temperature electrical insulation and aerospace structures, where dimensional stability under heat cycling determines long-term reliability.

### 3.6. Electrical Conductivity Testing

In this study, composite laminates with a [0]_10_ lay-up configuration were used. Electrical resistance tests were carried out on both neat and BN-reinforced specimens. For each specimen, eleven measurements were performed, and the average values obtained for each BN type and content are presented in Table 7.

Table 7 compares the average resistance values of composites containing different BN particle sizes (65–75 nm and 790 nm) and contents with those of the neat reference specimen, revealing distinct trends. According to Table 7, the addition of 0.2 wt.% BN increased the resistance slightly for both particle sizes compared to the neat specimen. However, at 0.4 wt.% content, a marked decrease in resistance was observed, particularly for the 790 nm BN specimen. This suggests that the particle size and content of BN not only influence the insulating behavior but may also affect microstructural orientation, potentially creating conductive pathways or interconnections that facilitate charge transport. These results are consistent with literature reports indicating the possibility of enhanced conductivity due to particle orientation effects.

Electrical resistance testing confirmed that h-BN largely preserved the insulating nature of the composites. Compared with neat laminates (~53 kΩ), 0.2 wt.% BN additions (both sizes) slightly increased resistance, while the 790 nm, 0.4 wt.% condition produced a modest decrease (~6.6%). These minor fluctuations suggest that BN does not intrinsically alter conductivity; rather, particle size and dispersion affect local morphology and potential leakage pathways.

The general preservation of high resistance aligns with BN’s wide band gap (~6 eV) and inherent insulating character. The modest reduction in the coarse/high-content case likely arises from microstructural bridging or surface leakage effects rather than any true conductive contribution. Similar behaviors have been noted in the literature, where BN fillers improved thermal conductivity while maintaining dielectric reliability [12,13].

From an application perspective, these findings highlight that BN enables multifunctional composites: mechanical and thermal improvements are achieved without compromising the electrical insulation required for aerospace and electronic encapsulation systems.

### 3.7. FTIR

For FTIR analysis, both neat and BN-reinforced specimens were prepared in powdered form. The spectra were plotted using Essential FTIR software (v3.50.227, Operant LLC, Racine, WI, USA).

FTIR analyses revealed the chemical structure and possible interfacial interactions in neat and BN-reinforced composites. In the neat specimen, the broad band observed around 3400 cm^−1^ corresponds to –OH stretching vibrations generated during epoxy curing, which are typically associated with hydrogen bonding and moisture uptake in epoxy systems [34,35]. The C–H stretching at 2925–2850 cm^−1^, the carbonyl (C=O) band at 1720 cm^−1^, the aromatic C=C band around 1600 cm^−1^, and the C–O–C stretching vibrations in the range of 1100–1250 cm^−1^ are characteristic features of epoxy, consistent with those reported in the literature [35].

**Figure 11:**
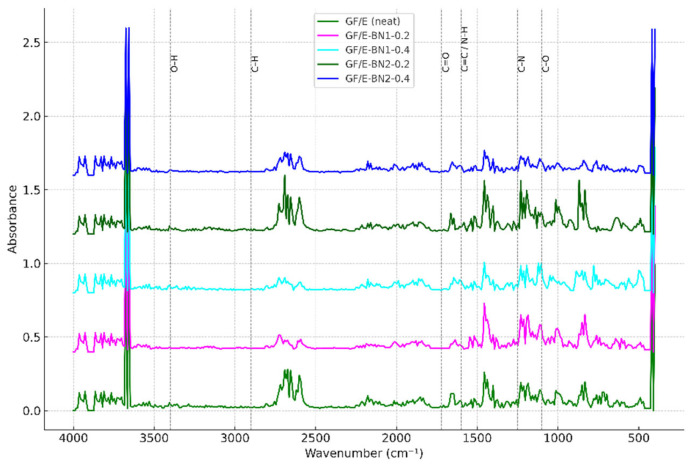
FTIR graph of neat and BN-reinforced E-glass/epoxy composites.

FTIR spectra provided direct evidence of filler–matrix interactions (Figure 11). Neat specimens exhibited characteristic epoxy absorptions (–OH stretching ~3400 cm^−1^, C=O at 1720 cm^−1^, C–O–C at 1100–1250 cm^−1^). With BN addition, these bands changed in both intensity and position, reflecting hydrogen bonding and van der Waals interactions between BN surface groups and epoxy chains. The effect was most evident at 0.2 wt.%, where increased intensity of the hydroxyl band indicated enhanced interfacial adhesion. At 0.4 wt.%, reduced sharpness of both BN (1380 cm^−1^, 810 cm^−1^) and epoxy bands suggested agglomeration and less homogeneous dispersion.

These results are consistent with prior studies where functionalized BN improved adhesion and shifted FTIR peaks [36,37]. The spectral evidence therefore complements the mechanical and thermal findings: low nanoscale BN contents enhance interfacial bonding, while higher loadings risk clustering and reduced interaction efficiency.

In practice, such chemical interactions underpin the observed improvements in tensile and shear strength, confirming that BN acts not merely as an inert filler but as an active interfacial modifier in the epoxy network.

## 4. Conclusions

In this study, glass fiber/epoxy laminates reinforced with hexagonal boron nitride (h-BN) at different particle sizes (65–75 nm and 790 nm) and weight fractions (0.2 and 0.4 wt.%) were systematically investigated. The findings demonstrated that h-BN significantly influenced the mechanical, thermal, and electrical properties of the composites, with the extent of improvement strongly dependent on particle size and filler content.

Mechanical tests demonstrated that the addition of 0.2 wt.% BN with a particle size of 65–75 nm provided the highest tensile and shear strengths, which were attributed to the homogeneous dispersion of nanoparticles and the strengthened fiber–matrix interface. In contrast, higher contents or larger particle sizes promoted agglomeration, leading to reduced strength. On the other hand, impact strength and hardness were markedly enhanced in the specimen reinforced with 0.4 wt.% BN at 790 nm, which can be explained by crack deflection and localized filler mechanisms.

Thermal analysis (DSC) showed that BN incorporation increased the glass transition temperature by restricting polymer chain mobility, thereby enhancing thermal stability. Electrical resistance measurements confirmed that h-BN maintained its intrinsic insulating character, with only minor fluctuations observed at higher contents of larger particles, likely due to morphological effects. FTIR spectra verified both the characteristic bands of epoxy and the presence of BN; moreover, the increased intensity of selected bands at low BN contents supported the existence of filler–matrix chemical interactions.

Overall, low contents of nanoscale BN were found to optimize tensile and shear performance, while larger particles provided advantages in terms of impact strength and surface hardness. These findings highlight that h-BN filler offers an effective pathway for the development of multifunctional composites. Future studies may further enhance these performances through surface modifications, hybrid filler strategies, or long-term environmental aging assessments.

The outcomes of this work demonstrate that h-BN reinforced glass fiber/epoxy laminates hold strong potential for applications in sectors such as aerospace, marine, and electrical insulation systems, where mechanical reliability, thermal stability, and dielectric performance are simultaneously required.

## Figures and Tables

**Table 1:** Specifications and typical mechanical properties of the E-glass fiber fabric used as reinforcement in this study (manufacturer data sheet).

Property	Value
Fiber type	E-glass fiber (unidirectional fabric)
Chemical composition	SiO_2_, CaO, B_2_O_3_, Al_2_O_3_, MgO, Fe_2_O_3_, R_2_O, BaO
Tensile strength	1400–1600 MPa
Tensile modulus	70–85 GPa
Density	2.60–2.65 g/cm^3^

**Table 2:** Nomenclature of the produced composite laminates.

Specimen Name	Abbreviation
E-glass fiber/epoxy (neat)	GF/E
E-glass fiber/epoxy + Boron nitride (65–75 nm-0.2 wt.%)	GF/E-BN1-0.2
E-glass fiber/epoxy + Boron nitride (65–75 nm-0.4 wt.%)	GF/E-BN1-0.4
E-glass fiber/epoxy + Boron nitride (790 nm-0.2 wt.%)	GF/E-BN2-0.2
E-glass fiber/epoxy + Boron nitride (790 nm-0.4 wt.%)	GF/E-BN2-0.4

**Table 3:** Tensile properties of neat and BN-reinforced E-glass/epoxy composites in fiber direction.

Composites	Tensile Strength (MPa)	Standard Deviation (%)	Elongation at Break (%)	Standard Deviation (%)	Elastic Modulus (GPa)	Standard Deviation (%)
GF/E	105.3	17.4	21.7	0.9	4.9	0.1
GF/E-BN1-0.2	337.4	39.1	8	0.7	21.8	1.5
GF/E-BN1-0.4	230.8	20.7	20.6	1.3	4.4	0.4
GF/E-BN2-0.2	85.9	0.3	7.1	0.1	21.7	1.6
GF/E-BN2-0.4	139.3	27.5	8.3	0.6	25.8	1

**Table 4:** Shear properties of neat and boron nitride-reinforced specimens.

Composites	Shear Strength (MPa)	Standard Deviation (%)	Elongation at Break (%)	Standard Deviation (%)	Shear Modulus (GPa)	Standard Deviation (%)
GF/E	35.1	0.8	7	0.1	5.2	0.4
GF/E-BN1-0.2	89.1	10.8	25.3	2.7	7.2	1
GF/E-BN1-0.4	73	1.2	20	1	6.8	0.4
GF/E-BN2-0.2	63.9	3.1	15.2	0.7	6.5	0.3
GF/E-BN2-0.4	58.3	1.1	17.2	1.4	6	0.6

**Table 5:** Impact properties of neat and boron nitride-reinforced specimens.

Composites	Mean Impact Strength (J/m)	Standard Deviation (%)
GF/E	24.5	2.1
GF/E-BN1-0.2	28	5.7
GF/E-BN1-0.4	42.3	2.5
GF/E-BN2-0.2	41.5	0.7
GF/E-BN2-0.4	53.3	3.1

**Table 6:** Hardness properties of neat and boron nitride-reinforced specimens.

Composites	Mean Hardness (HRB)	Standard Deviation (%)
GF/E	82.79	18.83
GF/E-BN1-0.2	87.59	11.1
GF/E-BN1-0.4	83.27	13.48
GF/E-BN2-0.2	87.42	16.2
GF/E-BN2-0.4	91.00	6.84

**Table 7:** Electrical conductivity properties of neat and boron nitride-reinforced specimens.

Composites	Mean Resistance (Ohm Ω)	Standard Deviation (%)
GF/E	53,444	6.1
GF/E-BN1-0.2	56,263	5.4
GF/E-BN1-0.4	58,483	7.4
GF/E-BN2-0.2	56,374	10.9
GF/E-BN2-0.4	49,916	8.4

## Data Availability

The original contributions presented in this study are included in the article and Supplementary Materials. Further inquiries can be directed to the corresponding author.

## Supplementary Materials

The following supporting information can be downloaded at: https://www.mdpi.com/article/10.3390/polym18030372/s1, Table S1: Raw experimental data of mechanical (tensile, in-plane shear, impact, and hardness), thermal (DSC), and electrical measurements of neat and BN-reinforced E-glass/epoxy laminated composites, obtained via the Susy system.
